# Atmospheric processes on ice nanoparticles in molecular beams

**DOI:** 10.3389/fchem.2014.00004

**Published:** 2014-02-24

**Authors:** Michal Fárník, Viktoriya Poterya

**Affiliations:** Laboratory of Molecular and Cluster Dynamics, Department of Ion and Cluster Chemistry, J. Heyrovský Institute of Physical Chemistry, Academy of Sciences of the Czech RepublicPrague, Czech Republic

**Keywords:** molecular beams, photodissociation, water clusters, atmospheric chemistry, aerosols, photochemistry, molecular dynamics

## Abstract

This review summarizes some recent experiments with ice nanoparticles (large water clusters) in molecular beams and outlines their atmospheric relevance: (1) Investigation of mixed water–nitric acid particles by means of the electron ionization and sodium doping combined with photoionization revealed the prominent role of HNO_3_ molecule as the condensation nuclei. (2) The uptake of atmospheric molecules by water ice nanoparticles has been studied, and the pickup cross sections for some molecules exceed significantly the geometrical sizes of the ice nanoparticles. (3) Photodissociation of hydrogen halides on water ice particles has been shown to proceed via excitation of acidically dissociated ion pair and subsequent biradical generation and H_3_O dissociation. The photodissociation of CF_2_Cl_2_ molecules in clusters is also mentioned. Possible atmospheric consequences of all these results are briefly discussed.

## 1. Introduction

The fact that small atmospheric ice particles and aerosols are important players in atmospheric chemistry has been recognized and outlined in textbooks, e.g., Finlayson-Pitts and Pitts (2000), and numerous review articles, e.g., Peter (1997); Ravishankara (1997); Finlayson-Pitts (2009); and Vaida (2011). Perhaps the most pronounced example is the stratospheric ozone depletion process, where the ice particles in polar stratospheric clouds (PSC) play a key role (Peter, 1997; Solomon, 1999; Prenni and Tolbert, 2001; Lu, 2010). The investigations of small nanometer-size particles directly in the atmosphere are difficult thus such studies are yet scarce, and large ambiguities exist in this size region. However, these small particles have large surface to volume ratio and offer a substantial playground for the surface assisted chemistry and photochemistry in the atmosphere. Recently Kulmala, et al. (2013) stressed the importance of the particles from this size range as the initial step in the atmospheric aerosol formation.

To investigate the relevant processes in laboratory, a vast number of studies on bulk ice surfaces are carried out including uptake and reactivity of atmospheric gasses on ice (Hanson, 1995; Oppliger et al., 1997; Huthwelker et al., 2006; Marcotte et al., 2013), and their photochemistry (Klán and Holoubek, 2002; Yabushita et al., 2002). On the other hand, the cluster physicists and chemists have developed a plethora of approaches for studying clusters in laboratory experiments in molecular beams which enable controlled generation of nanometer-size particles with composition corresponding to the atmospheric species (Campargue, 2001; Buch and Devlin, 2003). The details of ice nanoparticles generation in supersonic expansions has been recently investigated by Kim et al. (2004), Manka et al. (2012), and Li et al. (2013). The individual particles can be investigated under controlled conditions in vacuum by various means: e.g., ionization (electron, photon) and mass spectrometry (MacTaylor and Castleman, 2000; Lengyel et al., 2012b); infrared (IR) spectroscopy (Yacovitch et al., 2011; 2012; Preston et al., 2012; Fujii and Mizuse, 2013) or ultraviolet (UV) photodissociation experiments (Kreher et al. 1999; Li and Huber, 2001; Poterya, et al. 2007, 2008a; 2011; Ončák et al., 2008, 2011); particle (electron, photon, neutron) scattering (Heath et al., 2002; Kim et al., 2004; Manka et al., 2012); special methods such as sodium doping and subsequent spectroscopies (Bobbert et al., 2002; Yoder et al., 2011; Pradzynski et al., 2012). Such experiments provide unprecedented molecular-level insight into the small particle generation, their (photo)chemistry and (photo)physics and detailed dynamics of the processes on/in these particles. This in turn offers valuable data for understanding and modeling of atmospheric chemistry. Some of the methods of aerosol particle spectroscopy have been reviewed in recent book (Signorell and Reid, 2011).

Here we review some recent results of the molecular beam experiments with the ice nanoparticles performed mostly in our laboratory. Our versatile experiment allows to look at different aspects of the ice nanoparticle chemistry and physics: e.g., nucleation, pickup processes, and photochemistry. The purpose of this review is to summarize the different viewpoints outlined in the individually published articles to provide a new perspective for such experiments with ice nanoparticles, and also to discuss some possible direct consequences for atmospheric chemistry and physics. Wherever similar experiments exist in the literature, we attempt to review them briefly and outline our work in their context.

We will start with nucleation of nitric acid ice particles as revealed by special Na-doping method. Then we report about ice particle growth by pickup of molecules. Finally, we discuss UV photodissociation of atmospheric molecules on ice nanoparticles which is the major focus of our studies.

## 2. Materials and methods

**Figure 1:**
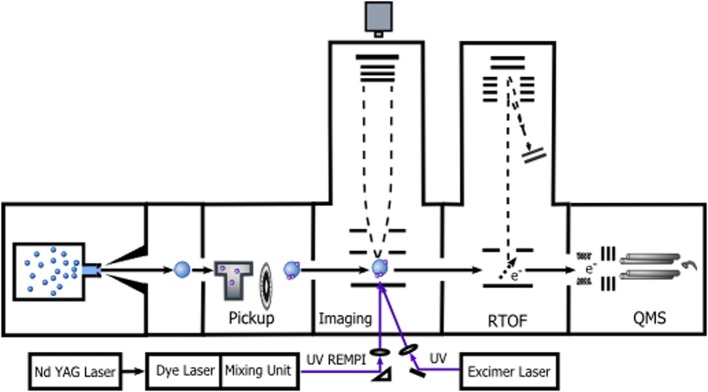
**Schematics of the experimental apparatus CLUB and the experiments outlined in this article**.

The majority of experiments reviewed in this article was performed in our laboratory on CLUster Beam apparatus (CLUB). A detailed description of CLUB setup can be found in Fárník (2011) and some recent extensions of this apparatus are outlined below. It is schematically depicted in Figure 1: it represents a unique and versatile setup for variety of experiments with clusters. The molecular beam is produced by a continuous supersonic gas expansion through a nozzle into vacuum. The mean cluster sizes can be controlled by the expansion conditions: pressure, temperature and nozzle geometry. The clusters can be doped with foreign molecules either in coexpansion or by passing them through a pickup cell filled with the molecular gas.

After passing through several differentially pumped high vacuum chambers the cluster beam enters the chamber where the photodissociation experiments are performed. The molecules in clusters are excited with nanosecond UV laser pulses. The desired photofragments are then selectively photoionized by resonance enhanced multiphoton ionization (REMPI) method and their kinetic energies are recorded. The time-of-flight (TOF) technique was used to measure the fragment velocity originally. Recently we have implemented the velocity map imaging (VMI). The ionized photofragments are detected with a large-area position sensitive detector with phosphor screen and the resulting images are recorded with a fast CCD camera. The image processing delivers a detailed 3D information about the photofragment velocities from which the photodissociation dynamics can be learned. More details about imaging and VMI techniques can be found in Chandler and Houston (1987); Eppink and Parker (1997); Gebhardt et al. (2001); and Whitaker (2003).

The next chamber along the beam path contains a new reflectron TOF mass spectrometer (RTOF) with electron and photo-ionization. It has been first implemented and described in our recent studies by Lengyel et al. (2012b, 2013), Kočišek et al. (2013a,b). At the end of the apparatus, the clusters can be also detected and analyzed by a quadrupole mass spectrometer after electron ionization. This option serves the beam alignment and analysis. Also cluster velocities can be measured using a (pseudorandom) chopper and the quadrupole detection to measure the cluster flight-times. Subsequently the cluster pickup cross sections can be evaluated from change in the cluster velocity in the pickup experiments. This method has been described by Fedor et al. (2011b).

Recently we have built another setup for studies of isolated molecules and small clusters: Apparatus for IMaging (AIM). Some of the results reviewed here were obtained on AIM. It implements the velocity mapping according to the design of Eppink and Parker (1997) and it has been described recently by Fedor et al., (2011a); Fárník et al. (2012); Poterya et al. (2014). We have built essentially identical VMI system for CLUB apparatus after testing it on AIM.

Several pulsed UV-laser systems are available in the laboratory: a fixed wavelength (193 nm) excimer laser; and two tunable systems (~200–400 nm) consisting of Nd:YAG lasers, dye lasers and frequency mixing units. In addition, a tunable IR OPO/OPA system can be also used. The laser beams from all these systems can be directed into different viewports in CLUB and AIM apparatus for the various experiments.

## 3. Results and discussion

### 3.1. HNO_3_ molecule as nucleation center

The PSC particles in the stratosphere can be pure water ice (type II), or ice containing nitric acid (type Ia), and nitric and sulphuric acids (type Ib) (Peter, 1997). To mimic these particles in laboratory experiments we have generated mixed water–nitric-acid clusters by nitric acid vapor expansions. Kayet al. (1981) first investigated these clusters by TOF mass spectrometry. Recently, Lengyel et al. (2012b) have characterized these clusters by mass spectrometry using two complementary ionization methods: (1) electron ionization and (2) photoionization after Na-doping. The analysis of the electron ionization mass spectra suggested that the second (and each subsequent) HNO_3_ molecule attaches more efficiently to the cluster when the previous HNO_3_ molecule(s) are already acidically dissociated: for example at least four water molecules are needed to dissociate an HNO_3_ molecule, therefore (HNO_3_)_2_·(H_2_O)*_n_* clusters with two HNO_3_ molecules appear for *n* ≥ 4–5 and have presumably the zwitterionic structure NO^−^_3_·H_3_O^+^·(H_2_O)_*n*− 1_·(HNO_3_). Analogically, the larger clusters with more HNO_3_ molecules are efficiently generated when all the previous HNO_3_ molecules in the cluster dissociate to ion pairs. These results were in accordance with the previous study by Kay et al. (1981). The Na-doping experiments below revealed another important property of mixed HNO_3_-water clusters: the HNO_3_ molecule is an effective nucleation center around which the clusters start to grow.

In electron ionization experiments generally the information about the neutral precursor clusters can be obtained rather indirectly from the mass spectra of the ionized fragments. To gain more insight into the nature of the neutral parent clusters the Na-doping method can be applied. This technique has been developed in Buck's laboratory (Bobbert et al., 2002; Zeuch and Buck, 2013) and used for size resolved infrared spectroscopy of large clusters (Forck et al., 2010; 2012; Pradzynski et al., 2012). Yoder et al. (2011) also proposed it as a general method for atmospheric aerosol detection and sizing (see also Forysinski et al., 2011). The experiments are based on the phenomena of solvated electron generation from Na in water clusters. The binding energy of the solvated electron (e.g., ~3 eV for water clusters) is significantly lower than the ionization potential of the cluster constituents. Therefore the clusters can be ionized with single low energy UV photon resulting in soft, essentially fragmentation-free, ionization. The measured mass spectrum after Na-pickup and photoionization thus reveals the original neutral cluster size distribution.

However, in our case of mixed (HNO_3_)*_m_*(H_2_O)*_n_* clusters no signal was detected after the Na doping: this observation contradicted the strong signals from the mixed cluster after electron ionization, and also the strong signals from the pure water clusters after the Na doping. The reason for this absence of signal from the mixed cluster turned out to be the fast charge transfer reaction: Na + HNO_3_ → Na^+^ + HNO^−^_3_. Therefore the cluster could not be ionized with the low energy photon as in the case of the solvated electron. Thus the Na-doping method as “the sizer for atmospheric aerosols” (Yoder et al., 2011) is limited to aerosols where sodium does not react with the constituent molecules.

This observation implied, that the nitric acid vapor expansions generated clusters which always contained at least one HNO_3_ molecule, i.e., no pure water clusters were formed.^1^ This in turn means that the HNO_3_ molecule acts as a very effective condensation nuclei. Could it play the same role in the atmosphere?

The stratospheric particles containing HNO_3_ molecules, i.e., type Ia PSCs are the most common ones, and thus determining for the ozone depletion. Yet, large uncertainties remain concerning their formation mechanism and composition (Peter, 1997; Finlayson-Pitts and Pitts, 2000; Prenni and Tolbert, 2001; Stetzer et al., 2006). The generally accepted models usually start with PSCs forming on background stratospheric aerosols composed primarily of sulphuric acid. Prenni and Tolbert (2001) outlined the interplay between nitric and sulphuric acids in PSCs. Although many studies concentrated on nitric acid aerosols, e.g., Dickens and Sloan (2002) and Stetzer, et al. (2006), the initial steps of small cluster formation have not been studied in detail. Our present findings suggest that the initial clusters can be efficiently formed around HNO_3_ molecule.

### 3.2. Particle growth and pickup cross sections

The atmospheric processes involving molecules on ice particles start by the pickup process in which the molecule lands on the particle. One of the quantities determining the further processes is the effective cross section of the ice nanoparticle for the pickup of the molecule. We can measure this quantity in our experiment by passing the cluster beam through a cell filled with a particular gas: the molecules collide with the clusters and are adsorbed, and the clusters are slowed down by these inelastic collisions. Cuvellier et al. (1991) demonstrated that by accurate measurements and analysis of cluster velocities after the pickup process the cluster mean sizes *N* could be determined. To calculate *N* they assumed that the pickup cross section corresponded to the geometrical one, σ*_g_*(*N*) = π *R*^2^_*N*_ (*R_N_* is the particle radius). This assumption can be tested, if the mean cluster size *N* is known (e.g., from other experiments). Then the effective pickup cross section σ(*N*) can be derived from the velocity measurements. We have carefully tested this method for the well studied argon clusters, compared it to other methods, and confirmed by theoretical simulations: (Fedor et al., 2011b) delivered reliable pickup cross sections for Ar*_N_* clusters.

Subsequently, Lengyel et al. (2012a) have applied this method to ice nanoparticles (H_2_O)*_N_* (*N* ≈ 260, *R_N_* ≈ 1.2Å). Pickup of various atmospherically relevant molecules has been measured, e.g., water, methane, NO_*x*_, hydrogen halides, and some volatile organic compounds. The water cluster mean sizes have been measured previously by Bobbert et al. (2002), therefore we could determine σ from the velocity measurements.

Our study has shown that the pickup cross sections can be significantly larger than the geometrical cross sections. Specifically σ ≈ 1000 Å^2^ for pickup of water molecules on (H_2_O)*_N_*, *N* ≈ 260, which is two-times larger than the geometrical cross section σ_*g*_ ≈ 500 Å^2^. The hexagonal ice density was used to calculate this geometrical cross section. Considering different densities of the cluster, the corresponding σ_*g*_ varied between 400 and 670 Å^2^. It is also worth mentioning that the above geometrical cross section is in agreement with theoretical calculations (Buch et al., 2004) which take into consideration real water–water potentials and hydrogen bonding in the cluster.

Our own molecular dynamics (MD) simulations were detailed in the corresponding publication (Lengyel et al., 2012a). The geometrical size of our simulated clusters was in agreement with the above σ_*g*_. Yet the cross section obtained from the MD simulations of the pickup processes on the clusters was 950 Å^2^ quite in agreement with the measured value. Thus the measured value has been confirmed by the MD simulations within the experimental error bars. More recently we have developed also a semiempirical analytical model which describes the measured cross sections. The polarizability of the molecule and clusters leads to the attraction of the molecules which collide with the clusters with scattering parameters significantly larger than the cluster radius resulting in the larger pickup cross section.

Our results can be compared to the measurements of pickup cross sections for protonated and deprotonated water clusters by Zamith et al. (2010a,b, 2013). In their experiments the charged clusters can be size selected and thus σ(*N*) for individual cluster sizes can be obtained. Our neutral cluster measurements are performed with the cluster size distributions produced in the expansions and are referred to the mean cluster size σ(*N*). Also the mass range of the experiments with ionic clusters is somewhat below our usual cluster sizes. Nevertheless, in the region where our data overlap, the agreement between the cross sections of ionic and neutral species is good (Zamith et al., 2013).^2^

More recently, we have investigated the size dependence of the pickup cross section. It turned out that the data depart even more from the geometrical value for the larger clusters *N* ≥ 300. In this region they exceed even the values from MD simulations. It appears that highly irregular particle shape for the larger clusters has to be invoked to explain these observations. There is an evidence that the larger clusters in supersonic expansions can grow by smaller cluster coagulation rather than by addition of individual molecules (Bobbert et al., 2002). This together with the directionality of the hydrogen bond in ice can give rise to highly irregular cluster shapes. We are currently working on theoretical simulations of this hypothesis.

It ought to be mentioned that the effective cross section is velocity dependent. We cannot lower the cluster beam velocity substantially to achieve the collision velocities corresponding to the real atmospheric temperatures (below 300 K). However, we can extrapolate the measured σ value using theoretical velocity dependence as outlined by Lengyel et al. (2012a). This extrapolation would yield σ≈ 1400 Å^2^ at the atmospherically relevant temperatures, i.e., cross section almost by factor of 3 larger than the geometrical value.

The atmospheric nucleation models generally assume the geometrical cross sections of spherical particles even for the small particles in nanometer-size range. For example recently Vehkamäki et al. (2012) have described a model which explicitly treats situations close to our experimentally studied case: the small cluster growth by monomer collisions with the cluster. The nucleation rates in their model are proportional to the collision coefficients which are taken to be the hard sphere collision rates. Thus the nucleation rates are proportional to the particle cross sections. Considering our experimental cross sections rather than the geometrical ones, the nucleation rates would be different by factor of 2–3. Our experiments have also shown that the cross sections differ for different molecules.

### 3.3. Photochemistry on nanoparticles

Large amount of attention has been paid to the photochemistry of hydrogen halides in water clusters due to their relevance to the ozone depletion process (Hurley et al., 2002, 2003; Dermota et al., 2005). Photodissociation in clusters has been also investigated for other relevant species, e.g., OClO (Fenner et al., 1997; Kreher et al., 1999), and nitric acid (Li and Huber, 2001). We concentrate on photodissociation dynamics of hydrogen halides and freons in/on large rare gas and water clusters.

#### 3.3.1. Hydrogen halides and hydronium radical

We investigated the HX (X = Cl, Br, I) photodissociation on (H_2_O)*_n_*, *n* ≈ 10^2^–10^3^, clusters to mimic the UV-photochemistry of hydrogen halides on ice particles in PSCs. The prerequisite for our present experiments were numerous previous studies of (HX)_*m*_·R*g_n_* clusters (Rg = Ar, Kr, Xe, Ne) performed originally in Buck's laboratory (Buck, 2002; Slavíček et al., 2003; 2004 Nahler et al., 2004a,b; Fárník et al., 2005; Fárník and Buck, 2006). The rare gas cluster represents a non-reactive solvent environment in which the HX photodissociation proceeds. The understanding of the photodissociation process in this inert solvent is necessary to gain some insight into the comparatively complex HX·(H_2_O)*_n_* system where chemistry can occur.

**Figure 2:**
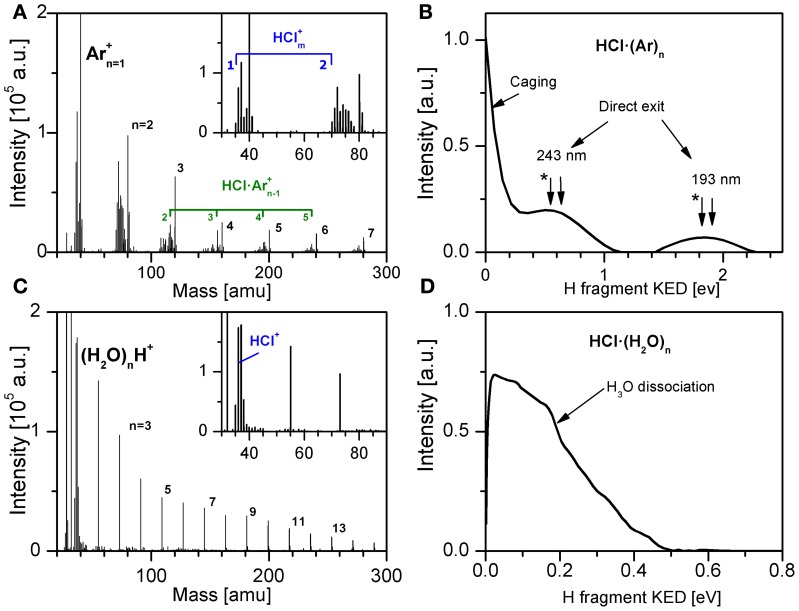
**Examples of experimental data measured for HCl pickup on Ar*_n_* (A,B), and HCl on (H_2_O)*_n_* (C,D): mass spectra (A,C) and H-fragment KED after the photodissociation with 193 nm (B,D)**. The KE corresponding to H fragment from free HCl molecule resulting in Cl^*^ spin-orbit excited state is labeled by ^*^ in **(B)**.

The rare gas experiments revealed “mechanistic effects” of photodissociating fragment: *caging* versus *direct exit*. These processes are expressed in the measured kinetic energy distributions (KED) of H-fragments as peaks at *zero kinetic energy* and at *energies corresponding to the photodissociation of the isolated molecule*, respectively, see Figure 2B. The vertical arrows label the energies corresponding to the photodissociation of isolated HCl molecule. The mass spectra Figure 2A serve not only to confirm the pickup of the molecule by the clusters, but also in case of multiple pickup the spectra reveal the dynamics of the molecules on the cluster surface and generation of small (HCl)*_m_* clusters. Despite the fact that the rare gas cluster represents an inert solvent environment the observed processes could be quite complex. For example Nahler et al. (2004a); Fárník and Buck (2006); and Poterya et al. (2008b) observed rare gas compounds HXeY (Y = I, Cl, C_2_H) generation and orientation. Therefore, detailed understanding of the rare gas systems facilitates the interpretation of the more complex results obtained with the water clusters below.

Large water clusters (H_2_O)*_n_*, *n* ≈ 10^2^–10^3^ were generated in our experiments and HX molecules were deposited on their surface in the pickup process discussed above. Figure 2C shows that the HCl molecules on the water cluster do not coagulate to HCl clusters as opposed to the Ar case shown in Figure 2A. An example of the H-fragment KED measured in several our studies (Poterya et al., 2007, 2008a; 2011; Ončák et al., 2008, 2011) is shown in Figure 2D. It exhibits some differences compared to the rare gas case: the direct exit fast fragments are missing entirely as well as the sharp peak at zero kinetic energy (cage effect); rather slow fragments extending to ≈0.5 eV occur with a broad maximum below 0.1 eV. These KEDs were invariant whether HCl, HBr or HI were adsorbed on (H_2_O)*_n_*, while they were distinctly different for these species on Rg*_n_* (the fast direct exit fragments reflect the different energetics of the corresponding HX molecule).

For the water–hydrogen halide system we have to consider the possibility of HX acidic dissociation on the cluster in its ground state. Do the UV-photons excite the covalently bound HX molecule on ice or H_3_O^+^·X^−^ ion pair structure? The experimental observation together with an earlier theoretical calculations of Sobolewski and Domcke (2002, 2003, 2007) lead to the proposal that the hydrogen atom originated from species with structure of *neutral hydronium radical* H_3_O. This hypothesis has been confirmed by experiments with deuterated species: namely, using HX·(H_2_O)*_n_*, DX·(H_2_O)*_n_*, and HX·(D_2_O)*_n_* and assuming the hydronium radical model, we expect to generate H_3_O, H_2_DO, and D_2_HO in these clusters, respectively. The expected H-atom signal ratio from these species then would be 3:2:1 (assuming the same clusters, expansion and pickup conditions, and observing only the H-signal, not D). This ratio has been confirmed for all three hydrogen halides, supporting the H_3_O hypothesis. The molecule was acidically dissociated on the ice nanoparticle and the ion pair structure was UV excited to a state of charge-transfer-to-solvent (CTTS) character where it relaxed to a biradical state where the hydrogen was released from H_3_O. It ought to be mentioned that subsequently there has been found an evidence for the H_3_O radical also in time dependent experiments of Hydutsky et al. (2009).

Interestingly, no significant H/D scrambling occurred in the clusters upon the acidic ionization and the H_3_O^+^·X^−^ ion pair remained of rather local nature. This can be clearly seen from the signal ratios: namely in HX·(H_2_O)*_n_* clusters with *n* ≈ 500, there is 10^3^-times more H-atoms than in HX·(D_2_O)*_n_*. If the H/D atoms in these clusters were all equivalent (statistical scrambling), there would be 10^3^-times higher probability to detect an H atom from HX·(H_2_O)*_n_* cluster than from HX·(D_2_O)*_n_*. However, the observed ratio of the signals from these two systems was only equal to 3 (not 10^3^) meaning that the H/D exchange did not occur within the clusters.

Our experiments complemented by theoretical investigations have delivered a clear picture of HX·(H_2_O)*_n_* photochemistry: HX first acidically dissociates, the system is photoexcited to a CTTS state, and Cl and H_3_O radicals are generated. Ončák et al. (2008, 2011) and Poterya et al., (2011) argued that the acidic dissociation in the ground state leads to a significant red-shift of the absorption spectra of the X^−^H_3_O^+^(H_2_O)_*n*−1_ species with possible atmospheric consequences.

There is more than three orders of magnitude increase between 150 and 320 nm (HCl and Cl_2_ absorption band maximum, respectively) in the actinic flux of photons in the stratosphere at ~50 km altitude. Therefore the ozone depletion models assume conversion of the reservoir species HCl to Cl_2_ on the ice particles in PSC. Subsequently, Cl_2_ is photolyzed to release Cl radicals into the stratosphere. However, the above mentioned red shift of the absorption spectra when HCl is adsorbed on the ice particles and acidically dissociated can significantly enhance the direct production of Cl radicals from photodissociation on ice particles (Ončák et al., 2008). Ončák et al., (2011) have outlined some questions which should be addressed in the future, namely: Should these direct processes on ice particles be included in the stratospheric ozone depletion models? How large is the spectral shift for particles of real atmospheric sizes? The shifts in our studies were calculated for fairly small clusters and they are actually smaller for the bulk ice. Will the Cl radicals leave the ice particles after the photodissociation or remain caged? We are currently working on some of these issues.

#### 3.3.2. Photochemistry of freon-12

**Figure 3:**
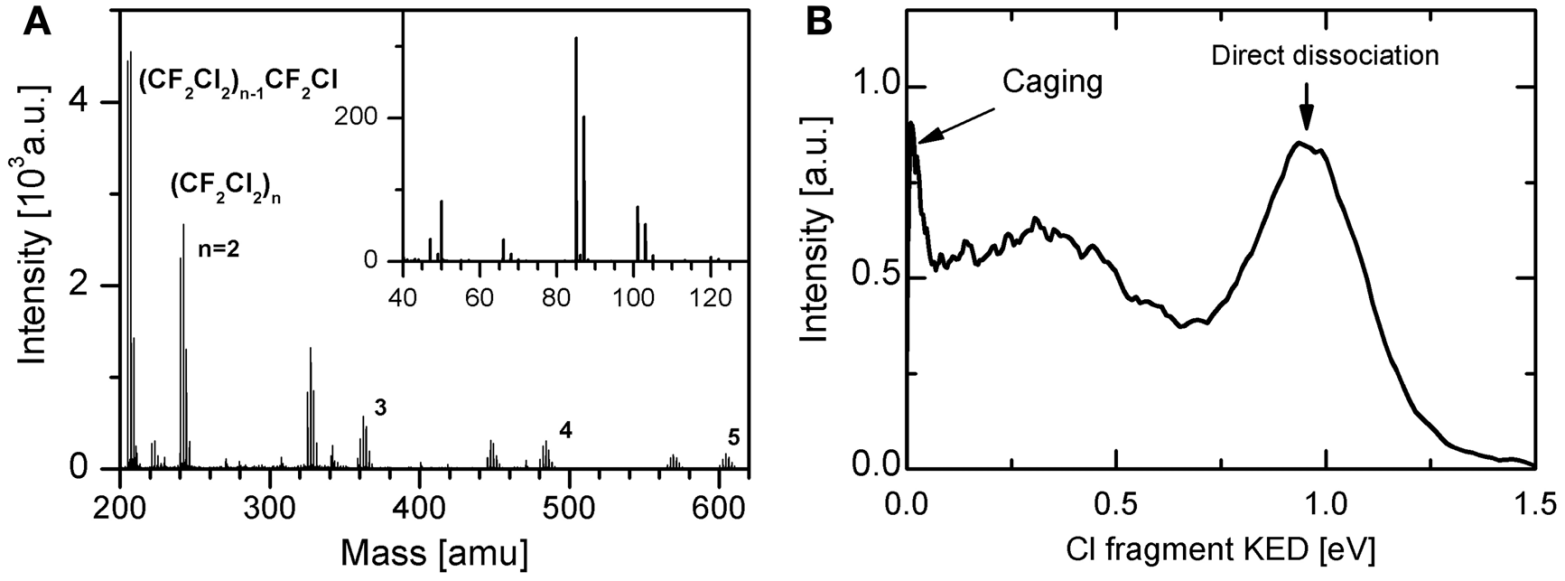
**Examples of experimental data measured for CF_2_Cl_2_/Ar system: electron ionization mass spectrum (A), and Cl-fragment KED after the photodissociation with 193 nm (B)**.

Recently, we have implemented the imaging and velocity mapping techniques for the photodissociation of molecules in clusters and Fedor et al. (2011a) tested it on the example of HBr photodissociation in rare gas clusters. With this new tool we have concentrated on another important atmospheric molecule, freon-12 CF_2_Cl_2_, relevant for stratospheric ozone depletion. In analogy to the hydrogen halides studies, Poterya et al. (2014) have first investigated the freon photodissociation dynamics in the environment of rare gas clusters. The mass spectrum in Figure 3A illustrates that freon molecules tend to generate (CF_2_Cl_2_)*_n_* clusters in large Ar*_N_* clusters. On the contrary no clusters were generated in pure freon expansions even at elevated stagnation pressures. The KEDs of Cl-fragments in different clusters exhibit caging and direct dissociation as illustrated in Figure 3B. Besides, the photodissociation dynamics of the bare CF_2_Cl_2_ molecule has been investigated by VMI technique in hitherto unreported details. Previous TOF studies (Baum and Huber, 1993; Yen et al., 1993) revealed some controversy concerning slow Cl fragments. Our VMI experiments revealed also some slower fragments in addition to the direct photodissociation yielding the fast fragments with kinetic energy *E*_kin_ ≈ 0.97 eV. Several processes were proposed to be responsible for these slow fragments, including concerted dissociation of two Cl atoms from single CF_2_Cl_2_ molecule.

There are two important results of our CF_2_Cl_2_ photodissociation experiments with possible consequences for atmospheric chemistry. The first one concerns the photodissociation of an isolated CF_2_Cl_2_ molecule. The observation of the slow Cl fragments which accounted for more than 20% of the total Cl signal cannot be explained by the direct fission of the C–Cl bond in CF_2_Cl_2_ molecule. Several pathways generating these slow fragments have been proposed by Poterya et al. (2014). They involve also concerted dissociation of two Cl atoms from the molecule, or secondary decay of an excited CF_2_Cl radical. Both these channels would yield two Cl atoms generated by one UV photon. Yet, the quantum yield measurements of Taketani et al. (2005) resulted in the quantum yield Φ = 1 at 193 nm. This value is also recommended for the atmospheric modeling (IUPAC). The proposed mechanisms leading to two Cl atoms released from one molecule upon single photon absorption does not have to be necessarily in discrepancy with the measured quantum yield of 1, if a compensating channel would exist where no Cl fragments were generated upon the photon absorption. The possibility of 2 Cl atoms released by a single UV photon could have consequences for the ozone hole modeling. However, it ought to be mentioned that the possibility of multiphoton processes (193 + 235 nm) could not be entirely excluded from our measurements as discussed by Poterya et al. (2014).

The second important result of the above study concerns the photodissociation in the clusters: it showed that part of the Cl fragments is caged totally in the cluster, while still significant fraction leaves the cluster undisturbed. Only in much larger clusters the probability of the direct exit from the cluster diminishes. The fragments which leave the cluster could contribute to the Cl budged in the atmosphere. Yet, it should be noted that the present experiments were performed for the model systems where the CF_2_Cl_2_ molecules were embedded inside the rare gas clusters. Currently we are performing the experiments with CF_2_Cl_2_ molecule deposited on the surface of large water clusters.

## 4. Summary

In summary, the present review shows several examples where detailed molecular level investigations of processes on nanoparticles in laboratory molecular beam experiments can assist understanding of atmospheric processes including ice and aerosol particles. The community of aerosol physicists and chemists has been mainly concentrated on the field measurements and large scale global modeling, while the molecular beam experiments have been focused on single molecules and small clusters. However, the message of the present review is to show that there is a large potential in overlapping these two areas, and that the molecular beams can offer unprecedented detailed insight even into the atmospherically relevant processes.

## Funding

Grant Agency of the Czech Republic, grants Nos.: 203/09/0422, P208/11/0161 and 14-08937S.

### Conflict of interest statement

The authors declare that the research was conducted in the absence of any commercial or financial relationships that could be construed as a potential conflict of interest.

## Footnotes

^1^Unless the nitric acid was diluted below concentrations of 1 mol% HNO_3_.

^2^Zamith et al. (2013) pointed out that the definition of the geometrical cross section σ_*g*_ = π × (*R_N_* + *r_m_*)^2^ should include the molecular radius *r_m_*. We have omitted *r_m_* to have the same σ_*g*_ for the water clusters when different molecules are being picked up by them. Considering this difference, both experiments agree with one another in the region where the data overlap.
